# Impact of number of critical care procedural skill repetitions on supervision level and teaching style

**DOI:** 10.1371/journal.pone.0280207

**Published:** 2023-01-23

**Authors:** Bjoern Zante

**Affiliations:** Department of Intensive Care Medicine, Inselspital, Bern University Hospital, University of Bern, Bern, Switzerland; Clinica Luganese Moncucco, SWITZERLAND

## Abstract

**Background:**

During critical care procedural skills training (e.g., in intubation and pericardiocentesis) the appropriate supervision level is important to ensure correct use of techniques and guarantee patient safety. The appropriate teaching style should be selected to address residents’ learning behavior and foster their competence. The aim of this study was to explore the number of repetitions for given skills needed to achieve a specified supervision level and a specific teaching style.

**Methods:**

This cross-sectional multicenter survey obtained data from residents and faculty of three multidisciplinary intensive care units (ICU) in Switzerland. Using a 4-point Likert scale, participants were asked to indicate the number of repetitions required to achieve the specified supervision level and teaching style.

**Results:**

Among 91 physicians, the response rate was 64% (n = 59). Their median estimations of the numbers of skill repetitions needed to achieve the final fourth level of supervision and final fourth stage of teaching style were as follows: arterial catheter insertion: supervision level 32, teaching style 17.5; peritoneal paracentesis: supervision level 27, teaching style 17; central venous catheter insertion: supervision level 38, teaching style 28; lumbar puncture: supervision level 38, teaching style 21; endotracheal intubation: supervision level 100, teaching style 45; chest drain insertion: supervision level 27, teaching style 21.5; temporary pacemaker placement: supervision level 50, teaching style 19.5; percutaneous tracheostomy: supervision level 50, teaching style 29; pericardiocentesis: supervision level 50, teaching style 35. Comparison of repetitions between supervision level and teaching style revealed no difference at the first and second levels, except for endotracheal intubation at level 2 (p = 0.03). Differences were observed at the third and fourth levels of supervision level and teaching style (p≤0.04).

**Conclusions:**

It appears that the supervision level and teaching style applied by faculty should change according to both the number of repetitions and the difficulty of critical care procedural skills.

## Introduction

Theory-driven concepts of medical education differ in their emphasis on the roles of learners (residents) and faculty in the process of learning/teaching [1–4]. Even the concept of competency-based medical education [5] and current critical care training programs pay insufficient attention to the interaction between residents and faculty [6–8]. However, it is particularly important that the appropriate supervision level is applied to ensure correct technique in the performance of skilled tasks and guarantee patient safety [9]. The teaching style should be adjusted according to residents’ individual learning behavior [10].

Depending on residents’ individual experience, different supervision levels may apply, as envisaged in ten Cate’s concept of entrustable professional activities independently of the time in training [11]. In brief, the different supervision levels are as follows: (1) practice skill under proactive, full supervision, as coactivity with faculty; (2) practice skill under reactive/on-demand supervision; (3) practice skill unsupervised; (4) resident supervises more junior colleagues [11].

**Fig 1:**
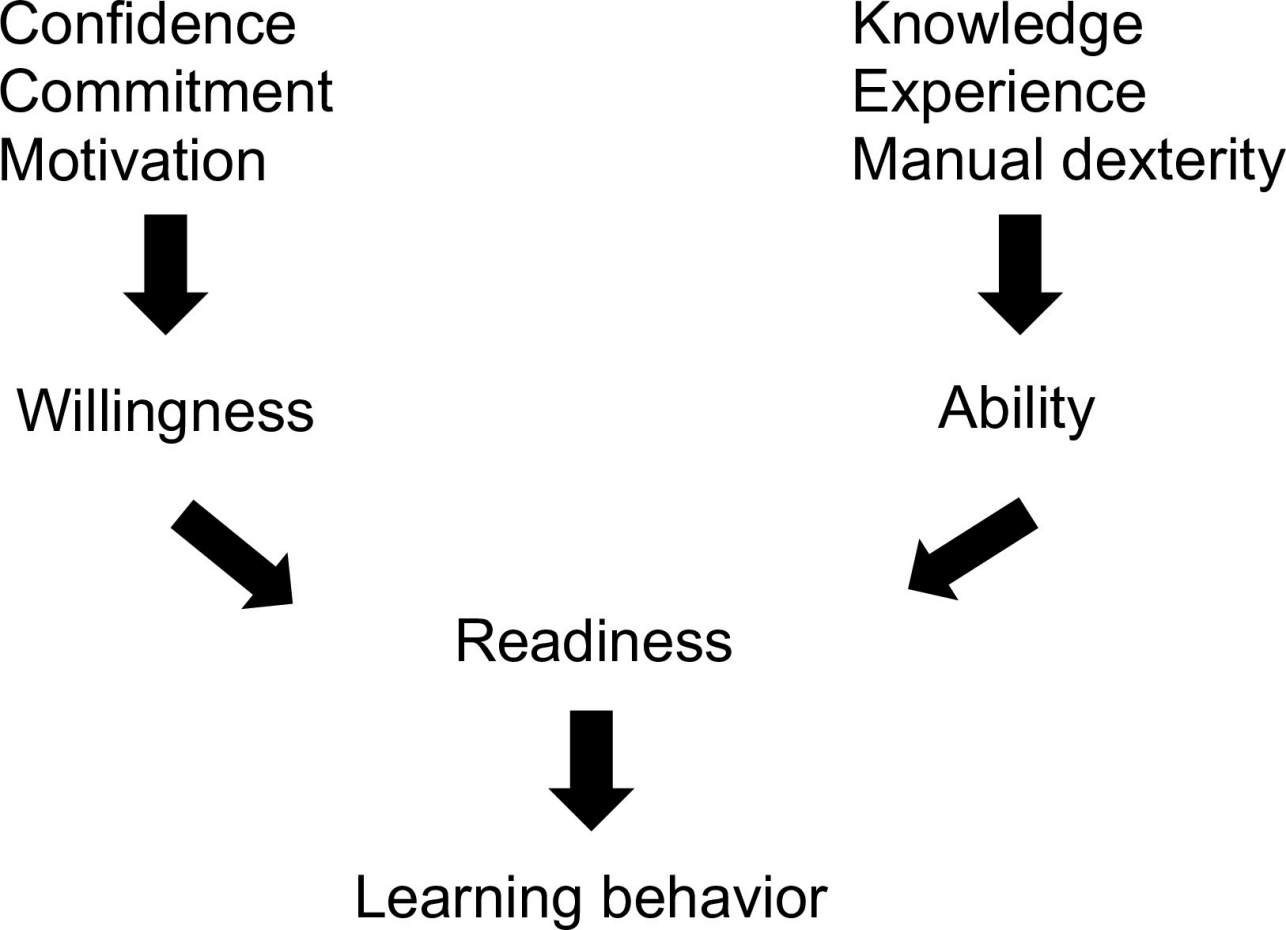
Learning behavior is determined by ability and willingness adapted from Hersey et al.’s Situational Leadership Theory [10,13].

The Situational Leadership Theory advanced by Hersey et al. (established in the mid-1970s) views effective leadership behavior as an “interplay among (1) the amount of guidance and direction a leader gives; (2) the amount of socioemotional support relationship behavior a leader provides; and (3) the readiness that individuals exhibit in performing a specific task” [12]. Based on this model, faculty should be able to adapt their teaching style to match the residents’ individual learning behavior, which in turn is dependent on the residents’ readiness (combination of ability and willingness; Fig 1).

Four different teaching styles: (1) directing style, (2) coaching style, (3) supporting style, and (4) delegating style are recommended depending on the individual learning behavior of the residents (Table 1) [13].

**Table 1:** Learning behavior and recommended teaching style.

Learning behavior	Teaching style
**Novice**	**Directing style**
• Not performing task to acceptable level • Being intimidated by task • Being unclear • Asking questions about task • Avoiding task or frustration • Being defensive or uncomfortable	• Detailed with incremental instructions (keep it simple and specific) • Providing specifics: who, what, when, where and how • Guiding, telling, directing • Predominantly one-way communication
**Advanced Beginner**	**Coaching style**
• Anxious or excited • Interested and responsive • Demonstrating moderate ability • Receptive to input • Attentive • Enthusiastic • New task, no experience	• Need for explaining decisions, and clarification • Providing specifics: who, what, when, where, how, and why • Reinforce small improvements • Two-way communication
**Competent**	**Supporting style**
• Demonstrated knowledge and ability • Appears hesitant to finish or take next step • Seems reluctant to perform alone • Solicits frequent feedback	• Support risk-taking, participating • Encouraging, supporting, empowering • Praise and build confidence • Two-way communication, active listening
**Proficient**	**Delegating style**
• Keeps teacher informed of task progress • Can operate autonomously • Is result-orientated • Shares both good and bad news • Makes effective decisions regarding task • Performs to high standards	• Need for delegating, observing, entrusting, assigning tasks • Support resident-made decisions • Reinforce results • Remain accessible

Definitions of learning behavior and recommended teaching styles adapted from Hersey et al.’s Situational Leadership Theory [10,13].

There is some evidence that the number of skill repetitions has an impact on supervision level and teaching behavior [9,10]. However, the data are limited [9,10,14,15].

This cross-sectional multicenter study was primarily conducted to assess the perception of critical care residents and the respective faculty as to what number of repetitions of critical care procedural skills is needed to achieve distinct supervision levels and for use of the appropriate teaching style. Secondary outcome measures were the individual experience regarding the critical care skills and the perceptions of the difficulty of the critical care skills and the comparison of the perceptions between residents and the faculty.

## Materials and methods

### Participants

The participants in this cross-sectional multicenter study were residents and faculty in multidisciplinary departments of intensive care medicine at three tertiary care teaching hospitals in Switzerland. The residents’ base specialties were anesthesiology, internal medicine, surgery (all subspecialties), special training in intensive care medicine, and others. Their duration of training in the ICU varied from 6 months or less to more than 12 months. All faculty members were specialists in intensive care medicine. All participants were sent an e-mail with an electronic link to the survey (S1 File).

### Survey

The survey was conducted using an online tool (UmfrageOnline, enuvo GmbH, Zurich, Switzerland). The following demographic indices were documented: for the residents, gender, postgraduate year (PGY), duration of training in the intensive care unit (ICU), and base specialty; for faculty, gender and PGY. Furthermore, the residents noted their experience (number of repetitions performed) in the following critical care skills: chest drain insertion, percutaneous tracheotomy, pericardiocentesis, endotracheal intubation, central venous catheter insertion, arterial catheter insertion, lumbar puncture, temporary pacemaker implantation, and peritoneal paracentesis. Experience was classified as number of skill repetitions: zero, 1–5, 6–10, 11–20, 21–50, or >50 repetitions.

Residents and faculty were asked to categorize specified critical care skills according to their difficulty. On the assumption of a wide range of difficulty of the skills, a 9-point Likert scale was used (e.g., insertion of an arterial catheter vs. pericardiocentesis).

The participants were also requested to estimate the number of repetitions required to achieve the following four supervision levels, defined according to ten Cate’s concept of entrustable professional activities [11]:

1. Practice skill under proactive, full supervision, as coactivity with faculty
2. Practice skill under reactive/on-demand supervision
3. Practice skill unsupervised
4. Allowed to supervise others

Finally, the participants indicated the number of repetitions required to achieve the specified perceived teaching style. Teaching styles were defined according to Hersey et al.’s Situational Leadership Theory (Table 1) [13].

### Ethics

The Ethics Committee of Bern (*Kantonale Ethikkommission Bern*) waived the need for ethics approval and the need to obtain consent for the collection, analysis, and publication of the data for this study (Req-2021-00079). Participation was voluntary and anonymous. This study adhered to the tenets of the Declaration of Helsinki.

### Statistical analysis

Statistical analysis was performed using MedCalc 17.4 (MedCalc Software, Ostend, Belgium). The demographic data were analyzed descriptively. The Spearman rank correlation test was used to compare residents’ and faculty’s classification of skill difficulty and the Mann-Whitney U-test to compare residents’ and faculty’s estimation of skill repetitions according to supervision level and teaching style. Furthermore, comparison of estimated skill repetitions for the supervision level and the level of teaching style was performed. For multiple testing and to control the type-I error rate at the nominal level of 0.05, we applied the Hochberg procedure to correct all p-values that were derived from different models [16]. A two-tailed p-value <0.5 was considered to indicate a significant difference.

## Results

Fifty-nine (64%) of 91 physicians responded to the survey. Thirty-three of the 59 participants (56%) were residents and 26 (44%) were faculty members. Overall, 97% (57/59) completed the assessment of skill difficulty, 80% (47/59) completed the assessment of supervision level, and 66% (38/59) completed the assessment of teaching behavior.

### Demographics

Forty-five percent (15/33) of the participating residents were female, 55% (18/33) male. The mean PGY was 6.76 years (standard deviation [SD] 2.44, range 3–14 years). The residents’ distribution among base disciplines and their duration of ICU training are shown in Table 2. The residents’ experience regarding skill performance is expressed as number of repetitions (Table 3). Forty-two percent (11/26) of the faculty were female, 58% (15/26) male. The mean PGY for the faculty was 12.76±4.82 years.

**Table 2:** Residents’ base disciplines and duration of intensive care training.

Base discipline						
Intensive care medicine	Anesthesiology		Internal medicine	Surgery		Other
12.1% (n = 4)	27.3% (n = 9)		39.4% (n = 13)	15.2% (n = 5)		6.1% (n = 2)
Duration of training in intensive care medicine						
≥ 6 months		7–12 months			>12 months	
39% (n = 13)		18% (n = 6)			42% (n = 14)	

**Table 3:** Number of skill repetitions performed by residents.

	Distribution of skill repetitions, % (n)					
	0	1–5	6–10	11–20	21–50	>50
Arterial catheter insertion	3% (1)	12% (4)	6% (2)	21% (7)	18% (6)	39% (13)
Peritoneal paracentesis	36% (12)	36% (12)	3% (1)	15% (5)	3% (1)	6% (2)
Central venous catheter insertion	9% (3)	6% (2)	15% (5)	21% (7)	15% (5)	33% (11)
Lumbar puncture	30% (10)	36% (12)	6% (2)	12% (4)	3% (1)	12% (4)
Endotracheal intubation	27% (9)	21% (7)	3% (1)	15% (5)	18% (6)	15% (5)
Chest drain insertion	24% (8)	39% (13)	18% (6)	18% (6)	0% (0)	0% (0)
Temporary pacemaker placement	36% (12)	36% (12)	3% (1)	15% (5)	3% (1)	6% (2)
Percutaneous tracheotomy	46% (15)	39% (13)	6% (2)	9% (3)	0% (0)	0% (0)
Pericardiocentesis	97% (32)	3% (1)	0% (0)	0% (0)	0% (0)	0% (0)

### Assessment of skill difficulty

The distribution of skill difficulty as assessed by residents and faculty is shown in Table 4. Spearman’s rank correlation indicated no differences between residents and faculty with regard to the ranking of skill difficulty: arterial catheter insertion: p = 0.68, 95% CI -0.35–0.16; central venous catheter insertion: p = 0.59, 95% CI -0.09–0.41; chest drain insertion: p = 0.91, 95% CI -0.23–0.29; endotracheal intubation: p = 0.59, 95% CI -0.13–0.38; lumbar puncture: p = 0.59, 95% CI -0.38–0.13; percutaneous tracheotomy: p = 0.59, 95% CI -0.41–0.1; pericardiocentesis: p = 0.59, 95% CI -0.11–0.4; peritoneal paracentesis: p = 0.69, 95% CI -0.34–0.18; temporary pacemaker placement: p = 0.94, 95% CI -0.27–0.25. Overall, residents and faculty rated the difficulty of these procedures in the following ascending order: (1) arterial catheter insertion, (2) peritoneal paracentesis, (3) central venous catheter insertion, (4) lumbar puncture, (5) endotracheal intubation, (6) chest drain insertion, (7) temporary pacemaker placement, (8) percutaneous tracheostomy, (9) pericardiocentesis.

**Table 4:** Distribution of skill difficulty ranking by residents and faculty.

	Skill difficulty								
	1	2	3	4	5	6	7	8	9
Arterial catheter insertion									
Residents	34% (11)	28% (9)	19% (6)	6% (2)	6% (2)	0% (0)	3% (1)	0% (0)	3% (1)
Faculty	36% (9)	40% (10)	16% (4)	4% (1)	0% (0)	0% (0)	0% (0)	4% (0)	0% (0)
Peritoneal paracentesis									
Residents	34% (11)	16% (5)	22% (7)	6% (2)	6% (2)	6% (2)	3% (1)	0% (0)	6% (2)
Faculty	48% (12)	12% (3)	8% (2)	4% (1)	16% (4)	4% (1)	0% (0)	4% (1)	4% (1)
Central venous catheter insertion									
Residents	16% (5)	28% (9)	19% (6)	25% (8)	0% (0)	13% (5)	0% (0)	0% (0)	0% (0)
Faculty	4% (1)	28% (7)	20% (5)	20% (5)	20% (5)	0% (0)	4% (1)	4% (0)	0% (0)
Lumbar puncture									
Residents	9% (3)	13% (4)	6% (2)	31% (10)	19% (6)	13% (4)	3% (1)	6% (2)	0% (0)
Faculty	0% (0)	12% (3)	36% (9)	28% (7)	12% (3)	4% (1)	4% (1)	0% (0)	4% (1)
Endotracheal intubation									
Residents	3% (1)	9% (3)	16% (5)	16% (5)	19% (6)	25% (8)	6% (2)	3% (1)	3% (1)
Faculty	4% (1)	4% (1)	8% (2)	24% (6)	12% (3)	20% (5)	20% (5)	4% (1)	4% (1)
Chest drain insertion									
Residents	0% (0)	3% (1)	9% (3)	9% (3)	25% (8)	25% (8)	22% (7)	6% (2)	0% (0)
Faculty	4% (1)	0% (0)	8% (2)	12% (3)	16% (4)	28% (7)	28% (7)	4% (1)	0% (0)
Temporary pacemaker placement									
Residents	0% (0)	0% (0)	6% (2)	6% (2)	19% (8)	9% (3)	38% (12)	9% (3)	13% (4)
Faculty	0% (0)	0% (0)	4% (1)	8% (2)	16% (4)	24% (6)	16% (4)	28% (7)	4% (1)
Percutaneous tracheostomy									
Residents	0% (0)	3% (1)	3% (1)	0% (0)	0% (0)	9% (3)	16% (5)	50% (16)	19% (6)
Faculty	4% (1)	0% (0)	0% (0)	0% (0)	8% (2)	8% (2)	24% (6)	48% (12)	8% (2)
Pericardiocentesis									
Residents	3% (1)	0% (0)	0% (0)	0% (0)	6% (2)	0% (0)	9% (3)	25% (8)	56% (18)
Faculty	0% (0)	4% (1)	0% (0)	0% (0)	0% (0)	12% (3)	4% (1)	4% (1)	76% (19)

Data expressed as % (n).

### Skill repetitions and supervision level

No differences were found between residents’ and faculty’s estimations of numbers of skill repetitions with regard to supervision level (S1 Table in S1 File).

Residents’ and faculty’s assessments of the number of repetitions required for the respective supervision levels are summarized in Table 5.

**Table 5:** Number of skill repetitions required to move up to next supervision level.

Skill	SL	Range	Median (95% CI)	IQR
Arterial catheter insertion	1	0–10	2 (1.9–3)	1–3
	2	1–20	5 (3–5)	3–10
	3	1–50	10 (8.9–10)	5–16.3
	4	0–200	15 (10–20)	10–25
Peritoneal paracentesis	1	0–5	2 (1.9–3)	1–5
	2	2–15	5 (5–5)	3–7
	3	2–30	10 (6.5–10)	5–15
	4	0–50	10 (10–15)	10–20
Central venous catheter insertion	1	0–10	3 (2.5–5)	2–5
	2	3–20	5 (5–10)	5–10
	3	2–30	10 (10–19.9)	10–20
	4	0–200	20 (13.1–23.7)	10–30
Lumbar puncture	1	0–10	3 (2–5)	1–5
	2	2–20	5 (5–10)	4–10
	3	2–50	10 (10–15)	20–10
	4	0–200	20 (13–20)	10–30
Endotracheal intubation	1	0–100	5 (5–10)	5–10
	2	2–250	15 (10–20)	10–30
	3	5–500	30 (20–53.9)	20–100
	4	0–1000	50 (33.8–100)	26.3–187.5
Chest drain insertion	1	0–10	2.5 (2–3)	2–5
	2	2–20	5 (5–5)	3–10
	3	2–50	10 (10–11.1)	5–15
	4	0–100	20 (15–20)	10–20
Temporary pacemaker placement	1	0–10	5 (3–5)	2–5
	2	2–20	10 (5–10)	5–12.5
	3	2–50	15 (10–17)	10–20
	4	0–100	20 (10–20)	10–33.8
Percutaneous tracheostomy	1	0–15	5 (3–5)	2–5
	2	2–20	10 (5–10)	5–15
	3	2–50	15 (10–20)	10–23.8
	4	0–150	20 (15.6–30)	10.5–40
Pericardiocentesis	1	0–20	5 (3–5)	2–10
	2	0–30	10 (5–10)	5–15
	3	2–50	15 (10–20)	10–26.3
	4	0–100	20 (10.4–29.1)	10–30

SL, Supervision level; 95% CI, 95% confidence interval; IQR, interquartile range.

### Skill repetitions and teaching style

No differences were found between residents’ and faculty’s estimations of numbers of skill repetitions with regard to teaching style (S2 Table in S1 File). Residents’ and faculty’s assessments of the number of repetitions required for respective teaching styles are summarized in Table 6.

**Table 6:** Number of skill repetitions required to move to next perceived teaching style.

Skill	TS	Range	Median (95% CI)	IQR
Arterial catheter insertion	1	0–30	2.5 (2–4)	1–5
	2	1–30	5 (3–5)	2–5
	3	1–30	5 (3–10)	3–10
	4	1–200	5 (5–10)	3–15
Peritoneal paracentesis	1	0–10	2 (2–5)	2–5
	2	1–10	5 (3–6)	2–10
	3	1–20	5 (3.5–8.5)	2–10
	4	1–50	5 (5–10)	2–10
Central venous catheter insertion	1	0–60	3 (2–5)	2–5
	2	1–60	5 (5–8.5)	3–10
	3	1–60	10 (5–10)	5–10
	4	1–200	10 (5–15)	5–20
Lumbar puncture	1	0–20	3.5 (2–5)	2–5
	2	1–20	5 (5–5)	4–8
	3	1–50	5 (5–10)	3–10
	4	1–200	7.5 (5–10)	3–15
Endotracheal intubation	1	0–100	5 (4.5–10)	3–15
	2	1–100	10 (6–13.5)	5–20
	3	1–100	15 (9.5–20)	5–30
	4	1–500	15 (6.5–30)	5–50
Chest drain insertion	1	0–200	3 (2–5)	2–5
	2	1–20	5 (3.9–5.2)	3–10
	3	1–30	5 (4.9–10)	3–10
	4	1–40	8.5 (5–10)	3–15
Temporary pacemaker placement	1	0–100	4.5 (3–10)	3–5
	2	2–100	5 (5–10)	5–10
	3	2–100	8.5 (5–10)	5–15
	4	1–100	9.5 (5–10)	5–20
Percutaneous tracheostomy	1	0–25	4.5 (3–5)	3–5
	2	2–25	5 (5–10)	4–10
	3	2–30	10 (5–10)	5–15
	4	1–50	10 (5–15)	5–20
Pericardiocentesis	1	0–30	5 (4–5.5)	3–10
	2	1–30	9 (5–10)	5–12.5
	3	2–30	10 (5–15)	5–15
	4	1–50	11 (8.3–16.7)	5–20

TS, Teaching style; 95% CI, 95% confidence interval; IQR, interquartile range.

### Comparison of repetitions for supervision level and level of teaching style

The estimated number of repetitions required did not differ at the first and second levels of supervision or for the directing and coaching styles of teaching (p-values are given in Table 7), with the exception of endotracheal intubation on the second level and the coaching style (p = 0.03; Table 7). Differences were observed for the third and fourth levels of supervision and the supporting and delegating styles of teaching (p-values are given in Table 7).

**Table 7:** Comparison of repetitions for supervision level and perceived teaching style.

Skill	SL	Teaching behavior	P-value
Arterial catheter insertion	1	Directing style	0.37
	2	Coaching style	0.37
	**3**	**Supporting style**	**0.004**
	**4**	**Delegating style**	**0.002**
Peritoneal paracentesis	1	Directing style	0.18
	2	Coaching style	0.52
	**3**	**Supporting style**	**0.004**
	**4**	**Delegating style**	**0.004**
Central venous catheter insertion	1	Directing style	0.93
	2	Coaching style	0.34
	**3**	**Supporting style**	**0.002**
	**4**	**Delegating style**	**0.003**
Lumbar puncture	1	Directing style	0.42
	2	Coaching style	0.37
	**3**	**Supporting style**	**0.001**
	**4**	**Delegating style**	**0.001**
Endotracheal intubation	1	Directing style	0.76
	**2**	**Coaching style**	**0.03**
	**3**	**Supporting style**	**0.001**
	**4**	**Delegating style**	**0.002**
Chest drain insertion	1	Directing style	0.1
	2	Coaching style	0.83
	**3**	**Supporting style**	**0.007**
	**4**	**Delegating style**	**0.004**
Temporary pacemaker placement	1	Directing style	0.37
	2	Coaching style	0.32
	**3**	**Supporting style**	**0.004**
	**4**	**Delegating style**	**0.008**
Percutaneous tracheostomy	1	Directing style	0.63
	2	Coaching style	0.07
	**3**	**Supporting style**	**0.003**
	**4**	**Delegating style**	**0.003**
Pericardiocentesis	1	Directing style	0.52
	2	Coaching style	0.79
	**3**	**Supporting style**	**0.02**
	**4**	**Delegating style**	**0.04**

Bold type indicates significant differences. SL, Supervision level.

## Discussion

In this cross-sectional multicenter study we explore associations between on the one hand the number of repetitions of critical care procedural skills and on the other hand supervision levels and teaching styles. Based on critical care residents’ and faculty’s ratings for cut-offs between one level/style and the next, potential learning curves were generated.

The level of supervision applied to ensure correct technique and guarantee patient safety may vary according to the residents’ experience of respective procedural skills. This experience, combined with acquired knowledge, attitudes, and values, may reflect the competence of the residents. In competency-based training it is essential to realize that the focus should be on individual performance rather than time in training, acknowledging that “high-performing” and “low-performing” individuals can both attain the required degree of competence. Importantly, simply performing a large number of skill repetitions does not guarantee competence; here, individual feedback is key to improve the residents’ performance. Furthermore, summative entrustment decisions are needed to guide the residents as they pass through the different supervision levels (levels 1–4) [17] and may leads to improved entrustment levels [18]. This could be done using appropriate checklists [19,20] with individual assistance from the faculty [21].

Besides the conventional bedside training, simulation training may be beneficial for common procedural skills (e.g., central venous catheter insertion) and particularly for less common skills (e.g., pericardiocentesis) [9,22,23].

Only sparse data are available on the numbers of skill repetitions needed to achieve particular supervision levels [14,24]. A recent research synthesis for certain skills (central venous catheter insertion, lumbar puncture, peritoneal paracentesis, and thoracocentesis) concluded that experience does not ensure competence. However, the number of skill repetitions deemed to represent clinical experience was very low (repetition categories 0, 1–2, 3–6, 7–10, 11–20, >21); this may explain why only 10% of the residents passed the assessments with a mean of 48% correct checklist items [25]. Other studies have observed that higher numbers of repetitions ensure safe skill performance [26,27]. Central venous catheter insertion by an experienced physician (≥50 catheterizations performed) is half as likely to result in complications compared with an inexperienced physician (<50 catheterizations) [26]. Physicians experienced in thoracocentesis (>30 repetitions) may perform better than their inexperienced colleagues (<30 repetitions) [27]. However, self-assessed confidence may rise in both experienced and inexperienced trainees after training [28].

Lower numbers of repetitions required for unsupervised execution have been suggested for peritoneal paracentesis [24]. These differences in numbers of repetitions for various skills could be explained by different degrees of skill difficulty, as observed in this study. However, even for more difficult skills such as pericardiocentesis, repetitions may lead to a reduced supervision level [9]. Therefore, the number of repetitions needed to ensure correct technique and guarantee patient safety may depend on skill complexity [29,30].

The recommended number of repetitions for endotracheal intubations in training varies from 33 to 200 [30–32]. As recently shown, the learning curve appears to plateau at 100–200 endotracheal intubations, and this is associated with a significant increase in the lowest oxygen saturation of patients undergoing intubation [33]. This seems to represent a good example of the importance of performing an appropriate number of repetitions of critical care procedural skills in order to achieve correct technique, establish a routine, and guarantee patient safety [34].

Faculty’s teaching style may change according to the residents’ learning behavior and is related to their state of readiness, which in turn depends on their ability and their willingness to perform a skill (Fig 1 and Table 1) [13]. The ability to perform a skill may relate to knowledge, experience, and manual dexterity. These factors may influence the cognitive load and vary directly with the mental effort and inversely the cognitive capacity to perform a skill or task [35,36]. Willingness may be related to residents’ confidence, commitment, and motivation (Fig 1) [37–39]. Both ability and willingness drive learning behavior, which in turn may drive the faculty’s teaching style (Table 1). Therefore, the faculty members can select the teaching style that will best foster the competence of the individual residents [40].

Interestingly, at the lower levels of supervision and teaching style for novices and advanced beginners, no differences in the estimated numbers of repetitions required were observed (except for endotracheal intubation) (Table 7). At levels 3 and 4, corresponding to advanced and proficient residents, higher numbers of repetitions were observed for supervision than for teaching style (Table 7). This could be interpreted as meaning that despite close supervision by the faculty members, their teaching style could be more relaxed. In turn, this may be explained by different purposes and progress of supervision and teaching style: while supervision is required to ensure patient safety and correct technique, faculty’s teaching style addresses residents’ learning behavior. Moreover, it is tempting to speculate that this might be affected by the potential unfamiliarity of the residents with respective skills. More than half (Table 2: internists, surgeons, and others) of the residents start their training in intensive care medicine without profound knowledge and routine regarding the critical care skills. This might lead to the demand for more repetitions, especially to attain the higher levels of supervision and teaching style.

### Limitations

This study has several noteworthy limitations. Because it was performed in only three ICUs in one country, the generalizability of its findings is limited. The small sample size and the low response rate mean that the results must be interpreted with caution (particularly in view of the high confidence intervals). Observational studies with larger sample sizes are needed to verify these findings. Furthermore, our data were self-assessed, with all the inherent limitations, and objective learning curves for respective skills must be generated. However, our findings may be useful for setting numbers of repetitions in future research on the supervision levels for critical care procedural skills. An additional focus should be on residents’ learning behavior and its underlying components, i.e., ability (knowledge, experience, and manual dexterity) and willingness (confidence, commitment, and motivation) with implications for the faculty’s teaching style.

## Conclusion

It appears that faculty’s supervision level and teaching style should be graduated according to the numbers of repetitions of critical care procedural skills. Knowledge of the association of numbers of skill repetitions with supervision levels and/or teaching styles may support both residents and faculty in learner-centered education.

## Supporting information

S1 File (DOCX)

## Abbreviations

SL: supervision level
TS: teaching style
ICU: intensive care unit
PGY: postgraduate year
CBME: competency-based medical education
EPA: entrustable professional activities
SD: standard deviation
CI: confidence interval

## References

[1] Levy IM, Pryor KW, McKeon TR. Is Teaching Simple Surgical Skills Using an Operant Learning Program More Effective Than Teaching by Demonstration? Clinical orthopaedics and related research. 2016;474(4):945–55. doi: 10.1007/s11999-015-4555-8 26369658 PMC4773331

[2] Willingham DB. A neuropsychological theory of motor skill learning. Psychological review. 1998;105(3):558–84. doi: 10.1037/0033-295x.105.3.558 9697430

[3] Shaker D. Cognitivism and psychomotor skills in surgical training: from theory to practice. International journal of medical education. 2018;9:253–4. doi: 10.5116/ijme.5b9a.129b 30269109 PMC6387771

[4] Kovacs G. Procedural skills in medicine: linking theory to practice. The Journal of emergency medicine. 1997;15(3):387–91. doi: 10.1016/s0736-4679(97)00019-x 9258796

[5] Long DM. Competency-based Residency Training. Academic Medicine. 2000;75(12):1178–83. 11112714 10.1097/00001888-200012000-00009

[6] Bion JF, Barrett H. Development of core competencies for an international training programme in intensive care medicine. Intensive care medicine. 2006;32(9):1371–83. doi: 10.1007/s00134-006-0215-5 16841214

[7] Bateman RM, Sharpe MD, Jagger JE, Ellis CG, Solé-Violán J, López-Rodríguez M, et al. 36th International Symposium on Intensive Care and Emergency Medicine: Brussels, Belgium. 15–18 March 2016. Critical care (London, England). 2016;20(Suppl 2):94. doi: 10.1186/s13054-016-1208-6 27885969 PMC5493079

[8] Barrett H, Bion JF. An international survey of training in adult intensive care medicine. Intensive care medicine. 2005;31(4):553–61. doi: 10.1007/s00134-005-2583-7 15750798

[9] Zante B, Schefold JC. Simulation training for emergency skills: effects on ICU fellows’ performance and supervision levels. BMC medical education. 2020;20(1):498. doi: 10.1186/s12909-020-02419-4 33298042 PMC7726897

[10] Zante B, Klasen JM. Learner-centered education: ICU residents’ expectations of teaching style and supervision level. BMC medical education. 2021;21(1):411. doi: 10.1186/s12909-021-02844-z 34330260 PMC8325219

[11] Ten Cate O. Nuts and bolts of entrustable professional activities. Journal of graduate medical education. 2013;5(1):157–8. doi: 10.4300/JGME-D-12-00380.1 24404246 PMC3613304

[12] Hersey P, Blanchard KH, Johnson DE. Management of Organizational Behavior: Leading Human Resources. Uttar Pradesh, India: Pearson; 2019.

[13] Hersey P, Blanchard KH, Johnson DE. Management of Organizational Behavior: Leading Human Resources. 10th ed. Upper Saddle River, N. J.: Pearson; 2012.

[14] Tariq M, Bhulani N, Jafferani A, Naeem Q, Ahsan S, Motiwala A, et al. Optimum number of procedures required to achieve procedural skills competency in internal medicine residents. BMC medical education. 2015;15:179. doi: 10.1186/s12909-015-0457-4 26493025 PMC4619250

[15] Fessler HE, Addrizzo-Harris D, Beck JM, Buckley JD, Pastores SM, Piquette CA, et al. Entrustable professional activities and curricular milestones for fellowship training in pulmonary and critical care medicine: report of a multisociety working group. Chest. 2014;146(3):813–34. doi: 10.1378/chest.14-0710 24945874

[16] Hochberg Y. A sharper Bonferroni procedure for multiple tests of significance. Biometrika. 1988;75(4):800–2.

[17] Ten Cate O, Schwartz A, Chen HC. Assessing Trainees and Making Entrustment Decisions: On the Nature and Use of Entrustment-Supervision Scales. Academic medicine: journal of the Association of American Medical Colleges. 2020;95(11):1662–9. doi: 10.1097/ACM.0000000000003427 32324633

[18] Valentine N, Wignes J, Benson J, Clota S, Schuwirth LW. Entrustable professional activities for workplace assessment of general practice trainees. The Medical journal of Australia. 2019;210(8):354–9. doi: 10.5694/mja2.50130 30977150

[19] McGaghie WC, Adams WH, Cohen ER, Wayne DB, Barsuk JH. Psychometric Validation of Central Venous Catheter Insertion Mastery Learning Checklist Data and Decisions. Simulation in Healthcare. 2021;16(6). doi: 10.1097/SIH.0000000000000516 33156260

[20] Mankute A, Juozapaviciene L, Stucinskas J, Dambrauskas Z, Dobozinskas P, Sinz E, et al. A novel algorithm-driven hybrid simulation learning method to improve acquisition of endotracheal intubation skills: a randomized controlled study. BMC Anesthesiology. 2022;22(1):42. doi: 10.1186/s12871-021-01557-6 35135495 PMC8822842

[21] Gaubert S, Blet A, Dib F, Ceccaldi P-F, Brock T, Calixte M, et al. Positive effects of lumbar puncture simulation training for medical students in clinical practice. BMC medical education. 2021;21(1):18. doi: 10.1186/s12909-020-02452-3 33407416 PMC7789333

[22] Jagneaux T, Caffery TS, Musso MW, Long AC, Zatarain L, Stopa E, et al. Simulation-Based Education Enhances Patient Safety Behaviors During Central Venous Catheter Placement. Journal of Patient Safety. 2021;17(6). doi: 10.1097/PTS.0000000000000425 28984729

[23] Okano H, Mayumi T, Kataoka Y, Banno M, Tsujimoto Y, Shiroshita A, et al. Outcomes of Simulation-Based Education for Vascular Access: A Systematic Review and Meta-Analysis. Cureus. 2021;13(8):e17188. doi: 10.7759/cureus.17188 34414052 PMC8365863

[24] Grabau CM, Crago SF, Hoff LK, Simon JA, Melton CA, Ott BJ, et al. Performance standards for therapeutic abdominal paracentesis. Hepatology (Baltimore, Md). 2004;40(2):484–8. doi: 10.1002/hep.20317 15368454

[25] Barsuk JH, Cohen ER, Feinglass J, McGaghie WC, Wayne DB. Residents’ Procedural Experience Does Not Ensure Competence: A Research Synthesis. Journal of graduate medical education. 2017;9(2):201–8. doi: 10.4300/JGME-D-16-00426.1 28439354 PMC5398145

[26] Sznajder JI, Zveibil FR, Bitterman H, Weiner P, Bursztein S. Central vein catheterization. Failure and complication rates by three percutaneous approaches. Archives of internal medicine. 1986;146(2):259–61. doi: 10.1001/archinte.146.2.259 3947185

[27] Rasmussen KMB, Hertz P, Laursen CB, Arshad A, Saghir Z, Clementsen PF, et al. Ensuring Basic Competence in Thoracentesis. Respiration. 2019;97(5):463–71. doi: 10.1159/000495686 30625480

[28] Spencer TR, Bardin-Spencer AJ. Pre- and post-review of a standardized ultrasound-guided central venous catheterization curriculum evaluating procedural skills acquisition and clinician confidence. The Journal of Vascular Access. 2019;21(4):440–8. doi: 10.1177/1129729819882602 31692399

[29] Nguyen BV, Prat G, Vincent JL, Nowak E, Bizien N, Tonnelier JM, et al. Determination of the learning curve for ultrasound-guided jugular central venous catheter placement. Intensive care medicine. 2014;40(1):66–73. doi: 10.1007/s00134-013-3069-7 23974524

[30] Buis ML, Maissan IM, Hoeks SE, Klimek M, Stolker RJ. Defining the learning curve for endotracheal intubation using direct laryngoscopy: A systematic review. Resuscitation. 2016;99:63–71. doi: 10.1016/j.resuscitation.2015.11.005 26711127

[31] Chichra A, Naval P, Dibello C, Tsegaye A, Mayo P, Koenig S, et al. Barriers to Training Pulmonary and Critical Care Fellows in Emergency Endotracheal Intubation Across the United States. Chest. 2011;140(4):1036A.

[32] Bernhard M, Mohr S, Weigand MA, Martin E, Walther A. Developing the skill of endotracheal intubation: implication for emergency medicine. Acta anaesthesiologica Scandinavica. 2012;56(2):164–71. doi: 10.1111/j.1399-6576.2011.02547.x 22060976

[33] Brown W, Janz DR, Russell D, Joffe EM, James DM, Vonderhaar DJ, et al. Effect of Operator Experience on Outcomes of Emergency Airway Management: The ICU Intubation Learning Curve. D25 CRITICAL CARE: HARD TIMES—RESUSCITATING MY PATIENT: FLUID, BLOOD AND OTHER STRATEGIES. American Thoracic Society International Conference Abstracts: American Thoracic Society; 2019. p. A5985-A.

[34] Sheng AY, Clark A, Amanti C. Supervision of Resident Physicians. Emergency Medicine Clinics of North America. 2020;38(2):339–51. doi: 10.1016/j.emc.2020.02.004 32336329

[35] Young JQ, Van Merrienboer J, Durning S, Ten Cate O. Cognitive Load Theory: implications for medical education: AMEE Guide No. 86. Medical teacher. 2014;36(5):371–84. doi: 10.3109/0142159X.2014.889290 24593808

[36] Sweller J, Ayres P, Klayuga S. Cognitive Load Theory. New York: Springer-Verlag; 2012.

[37] Taylor DCM, Hamdy H. Adult learning theories: Implications for learning and teaching in medical education: AMEE Guide No. 83. Medical teacher. 2013;35(11):e1561–e72. doi: 10.3109/0142159X.2013.828153 24004029

[38] Lebreton M, Bacily K, Palminteri S, Engelmann JB. Contextual influence on confidence judgments in human reinforcement learning. PLoS computational biology. 2019;15(4):e1006973. doi: 10.1371/journal.pcbi.1006973 30958826 PMC6472836

[39] Pelaccia T, Viau R. Motivation in medical education(). Medical teacher. 2017;39(2):136–40. doi: 10.1080/0142159X.2016.1248924 27866457

[40] Nieboer P, Huiskes M. The regulation of learning in clinical environments: A comment on ’Beyond the self’. Medical education. 2020;54(3):179–81. doi: 10.1111/medu.14055 31912557 PMC7065238

