# Supplementary material for: Impact of number of critical care procedural skill repetitions on supervision level and teaching style
Source: PLoS One. 2023 Jan 23;18(1):e0280207. doi: 10.1371/journal.pone.0280207 (PMC9870148; doi:10.1371/journal.pone.0280207)
Supplement: S1 File — (DOCX) [file pone.0280207.s001.docx]

**Procedural critical care skills: optimum number for supervision levels and teaching style**

Bjoern Zante MD MME^1^

1. Department of Intensive Care Medicine, Inselspital, Bern University Hospital, University of Bern, Bern, Switzerland

**Corresponding author:**

Dr. med. Bjoern Zante, MD MME

Department of Intensive Care Medicine

Bern University Hospital

Freiburgstrasse 10, 3010 Bern, Switzerland

Fax: +41 (0)31 632 17 71

Phone: +41 (0)31 632 53 00

E-mail: bjoern.zante@insel.ch

**Supplemental**

**Questionnaire**

**Table S1.** Number of skill repetitions for regarding supervision

**Table S1.** Number of skill repetitions for regarding supervision level

| Skill | SL | Participant | Range | Median  (95%-CI) | IQR | p-value |
| --- | --- | --- | --- | --- | --- | --- |
| Arterial line insertion | 1 | Residents | 0-10 | 2 (1-3) | 1-3 | 0.22 |
|  |  | Faculty | 1-5 | 3 (2-4.2) | 2-5 |  |
|  | 2 | Residents | 1-20 | 5 (3-10) | 3-10 | 0.91 |
|  |  | Faculty | 2-15 | 5 (3-6.2) | 3-7 |  |
|  | 3 | Residents | 1-50 | 10 (10-20) | 6.25-20 | 0.22 |
|  |  | Faculty | 2-20 | 9 (5-10) | 5-10 |  |
|  | 4 | Residents | 1-200 | 15 (10-25) | 10-26.25 | 0.52 |
|  |  | Faculty | 0-200 | 12.5 (8.8 – 20) | 8-20 |  |
| Peritoneal paracentesis | 1 | Residents | 0-5 | 3 (1-3) | 1-4 | 0.91 |
|  |  | Faculty | 0-5 | 2 (1-4.2) | 1-5 |  |
|  | 2 | Residents | 2-15 | 5 (5-5) | 4-10 | 0.29 |
|  |  | Faculty | 2-10 | 5 (2.4-5) | 2-5 |  |
|  | 3 | Residents | 3-30 | 10 (10-15) | 10-15 | 0.09 |
|  |  | Faculty | 2-15 | 6 (5-10) | 5-10 |  |
|  | 4 | Residents | 5-50 | 15 (10-20) | 10-25 | 0.22 |
|  |  | Faculty | 0-50 | 10 (5-18) | 5-20 |  |
| CV line insertion | 1 | Residents | 0-10 | 3 (2-5) | 2-5 | 0.71 |
|  |  | Faculty | 1-7 | 3.5 (2.4 – 5) | 2-5 |  |
|  | 2 | Residents | 2- 20 | 7.5 (5-10) | 5-10 | 0.52 |
|  |  | Faculty | 3-12 | 5 (5-10) | 5-10 |  |
|  | 3 | Residents | 3-30 | 15 (10-20) | 10-20 | 0.22 |
|  |  | Faculty | 2-25 | 10 (10-16.2) | 10-17 |  |
|  | 4 | Residents | 10-200 | 20 (10.7-29.4) | 10-30 | 0.87 |
|  |  | Faculty | 0-200 | 20 (10-30) | 10-30 |  |
| Lumbar puncture | 1 | Residents | 0-15 | 3 (2-8.26) | 1.25-10 | 0.71 |
|  |  | Faculty | 1-20 | 4 (2.3-5) | 2.3-5 |  |
|  | 2 | Residents | 1-20 | 5 (5-6.95) | 4.25-9.5 | 0.98 |
|  |  | Faculty | 2-20 | 5 (3.3-7.7) | 3.25-7.8 |  |
|  | 3 | Residents | 1-50 | 5 (4.4-10) | 3.3-10 | 0.71 |
|  |  | Faculty | 3-20 | 5 (3.3-10) | 3.25-10 |  |
|  | 4 | Residents | 1-50 | 7.5 (3-10) | 3-10 | 0.6 |
|  |  | Faculty | 3-200 | 7.5 (4.6-15) | 4.5-15 |  |
| Endotracheal intubation | 1 | Residents | 0-25 | 5 (5-9.28) | 3-10 | 0.22 |
|  |  | Faculty | 1-100 | 10 (5-18) | 5-20 |  |
|  | 2 | Residents | 2-100 | 15 (10-23.2) | 10-30 | 0.46 |
|  |  | Faculty | 5-250 | 20 (10-42.1) | 10-50 |  |
|  | 3 | Residents | 10-200 | 30 (20-50) | 20-60 | 0.83 |
|  |  | Faculty | 5-500 | 40 (20-100) | 20-100 |  |
|  | 4 | Residents | 10-1000 | 50 (32.4-100) | 30-162.5 | 0.94 |
|  |  | Faculty | 0-1000 | 75 (21.99-160.26) | 20-200 |  |
| Chest drain insertion | 1 | Residents | 0-5 | 2 (2-3) | 1-5 | 0.47 |
|  |  | Faculty | 0-10 | 3 (2-5) | 2-5 |  |
|  | 2 | Residents | 2-20 | 5 (5-5.1) | 5-9 | 0.52 |
|  |  | Faculty | 2-10 | 4.5 (3-8.4) | 3-10 |  |
|  | 3 | Residents | 3-50 | 10 (10-15.2) | 10-20 | 0.22 |
|  |  | Faculty | 2-20 | 9 (5-13) | 5-15 |  |
|  | 4 | Residents | 5-100 | 20 (15-20) | 12-25 | 0.37 |
|  |  | Faculty | 0-40 | 17.5 (5.4-20) | 5-20 |  |
| Temp. pacemaker placement | 1 | Residents | 0-10 | 5 (3-5) | 2-5 | 0.71 |
|  |  | Faculty | 0-10 | 3.5 (2-5) | 2-5 |  |
|  | 2 | Residents | 2-20 | 10 (5-15) | 5-15 | 0.15 |
|  |  | Faculty | 2-20 | 5 (4.4-10) | 4-10 |  |
|  | 3 | Residents | 5-50 | 15 (10-20) | 10-25 | 0.09 |
|  |  | Faculty | 2-30 | 10 (6.2-15) | 5-15 |  |
|  | 4 | Residents | 9-100 | 20 (10.7) | 10-35 | 0.22 |
|  |  | Faculty | 0-50 | 12.5 (7-20) | 5-20 |  |
| Percutaneous tracheotomy | 1 | Residents | 0-15 | 5 (2.4-5) | 2-5 | 0.98 |
|  |  | Faculty | 1-10 | 4 (2.4-5) | 2-5 |  |
|  | 2 | Residents | 5-20 | 10 (6-15) | 5-15 | 0.11 |
|  |  | Faculty | 2-20 | 5 (5-10) | 5-10 |  |
|  | 3 | Residents | 5-50 | 20 (15-25) | 10-26.5 | 0.09 |
|  |  | Faculty | 2-50 | 10 (8.8-13.8) | 8-15 |  |
|  | 4 | Residents | 5-150 | 30 (20-39.3) | 20-50 | 0.09 |
|  |  | Faculty | 0-70 | 15 (8.8-23) | 8-25 |  |
| Pericardio-centesis | 1 | Residents | 0-15 | 5 (3.7-10) | 2-10 | 0.67 |
|  |  | Faculty | 0-20 | 4.5 (2-8) | 2-10 |  |
|  | 2 | Residents | 0-30 | 10 (5.2-14.3) | 5-16.3 | 0.22 |
|  |  | Faculty | 2-25 | 5 (4-10) | 3.75-10 |  |
|  | 3 | Residents | 5-50 | 20 (10-22.5) | 10-30 | 0.22 |
|  |  | Faculty | 2-30 | 10 (6-20) | 5.8-21.3 |  |
|  | 4 | Residents | 5-100 | 20 (13.7-30) | 10-32.5 | 0.04 |
|  |  | Faculty | 0-50 | 13.5 (7.6-25) | 6-25 |  |

SL: supervision level, 95%-CI: 95% confidence interval, IQR interquartile range

**Table S2.** Number of skill repetitions for regarding teaching style

| Skill | TS | Participant | Range | Median  (95%-CI) | IQR | p-value |
| --- | --- | --- | --- | --- | --- | --- |
| Arterial line insertion | 1 | Residents | 0-15 | 2 (1-4.3) | 1-5 | 0.97 |
|  |  | Faculty | 1-30 | 3 (2-5) | 2-5 |  |
|  | 2 | Residents | 1-15 | 5 (2.4-5) | 2-5 | 0.97 |
|  |  | Faculty | 1-30 | 5 (2.3-6.5) | 2.3-6.5 |  |
|  | 3 | Residents | 1-20 | 5 (2-10) | 2-10 | 0.97 |
|  |  | Faculty | 1-30 | 5 (3-10) | 3-10 |  |
|  | 4 | Residents | 1-20 | 5 (3-10) | 2-10 | 0.97 |
|  |  | Faculty | 1-200 | 7 (5-15) | 5-15 |  |
| Peritoneal paracentesis | 1 | Residents | 0-10 | 2 (2-5) | 2-5 | 0.97 |
|  |  | Faculty | 1-10 | 3 (2-5) | 2-5 |  |
|  | 2 | Residents | 1-10 | 5 (3-6.3) | 2.3-9.3 | 0.97 |
|  |  | Faculty | 2-10 | 5 (2-9.5) | 2-9.5 |  |
|  | 3 | Residents | 1-20 | 5 (2.4-10) | 2-10 | 0.97 |
|  |  | Faculty | 1-15 | 5 (3.3-10) | 3.3-10 |  |
|  | 4 | Residents | 1-30 | 5 (2-10) | 2-10 | 0.97 |
|  |  | Faculty | 1-50 | 5.5 (5-11.8) | 5-12.5 |  |
| CV line insertion | 1 | Residents | 0-15 | 2 (1.4-5) | 1-5 | 0.97 |
|  |  | Faculty | 1-60 | 4 (2-5) | 2-5 |  |
|  | 2 | Residents | 1-15 | 5 (2.4-8.3) | 2-10 | 0.97 |
|  |  | Faculty | 2-60 | 5 (5-10) | 5-10 |  |
|  | 3 | Residents | 1-20 | 10 (3.1-10) | 2-10 | 0.97 |
|  |  | Faculty | 2-60 | 10 (5-13.7) | 5-13.8 |  |
|  | 4 | Residents | 1-50 | 5 (3-10.2) | 2-15 | 0.97 |
|  |  | Faculty | 3-200 | 12.5 (5-20) | 5-20 |  |
| Lumbar puncture | 1 | Residents | 0-15 | 3 (2-8.3) | 1.3-10 | 0.97 |
|  |  | Faculty | 1-5 | 3 (1.4-5) | 1-5 |  |
|  | 2 | Residents | 1-20 | 5 (5-7) | 4.3-9.5 | 0.99 |
|  |  | Faculty | 2-20 | 5 (3.3-7.8) | 3.3-7.8 |  |
|  | 3 | Residents | 1-50 | 5 (4.4-10) | 3.3-10 | 0.97 |
|  |  | Faculty | 3-20 | 5 (3.3-10) | 3.3-10 |  |
|  | 4 | Residents | 1-50 | 7.5 (3-10) | 3-10 | 0.97 |
|  |  | Faculty | 3-200 | 7.5 (4.6-15) | 4.5-15 |  |
| Endotracheal intubation | 1 | Residents | 0-100 | 5 (3-10) | 2.3-13.8 | 0.97 |
|  |  | Faculty | 2-90 | 5 (2.3-13.8) | 3.3-13.8 |  |
|  | 2 | Residents | 1-100 | 10 (5-10) | 5-13.75 | 0.97 |
|  |  | Faculty | 3-90 | 12 (5.5-20) | 5.5-20 |  |
|  | 3 | Residents | 1-100 | 10 (5-20) | 5-27.5 | 0.97 |
|  |  | Faculty | 4-90 | 15 (6.3-37.4) | 6.3-37.5 |  |
|  | 4 | Residents | 1-100 | 9.5 (5-30) | 5-30 | 0.97 |
|  |  | Faculty | 4-500 | 20 (8.2-64.5) | 7.5-70 |  |
| Chest drain insertion | 1 | Residents | 0-15 | 3 (2-5) | 2-5 | 0.97 |
|  |  | Faculty | 1-20 | 4 (2-5.4) | 2-5.5 |  |
|  | 2 | Residents | 2-15 | 5 (3-5) | 3-8.8 | 0.97 |
|  |  | Faculty | 1-20 | 5 (2.6-10) | 2.4-10 |  |
|  | 3 | Residents | 1-20 | 5 (3.5-10) | 3-10 | 0.97 |
|  |  | Faculty | 1-30 | 6.5 (3.6-10) | 3.5-10 |  |
|  | 4 | Residents | 1-25 | 6.5 (3-15 | 3-15 | 0.97 |
|  |  | Faculty | 1-40 | 10 (5.13.1) | 5-13.5 |  |
| Temp. pacemaker placement | 1 | Residents | 0-100 | 4 (3-8.3) | 2.3-10 | 0.99 |
|  |  | Faculty | 1-20 | 5 (3-5) | 3-5 |  |
|  | 2 | Residents | 2-100 | 5 (5-10) | 4.3-10 | 0.97 |
|  |  | Faculty | 2-20 | 5 (5-10) | 5-10 |  |
|  | 3 | Residents | 2-100 | 7 (5-15) | 5-18.8 | 0.97 |
|  |  | Faculty | 2-15 | 10 (5-10) | 5-10 |  |
|  | 4 | Residents | 1-100 | 8.5 (4.9-20) | 3-20 | 0.97 |
|  |  | Faculty | 2-50 | 10 (5-15) | 5-15 |  |
| Percutaneous tracheotomy | 1 | Residents | 0-25 | 5 (3-5) | 2.3-8.8 | 0.99 |
|  |  | Faculty | 2-20 | 4 (3-5) | 3-5 |  |
|  | 2 | Residents | 2-25 | 5 (5-10) | 3.5-10 | 0.99 |
|  |  | Faculty | 3-15 | 6 (4.3-10) | 4.3-10 |  |
|  | 3 | Residents | 2-30 | 10 (5-15) | 5-18.8 | 0.97 |
|  |  | Faculty | 3-15 | 10 (4.3-10) | 4.3-10 |  |
|  | 4 | Residents | 1-50 | 10 (4.9) | 3-20 | 0.99 |
|  |  | Faculty | 3-50 | 10 (5-13.1) | 5-13.5 |  |
| Pericardio-centesis | 1 | Residents | 0-20 | 5 (3.4-10) | 2.3-10 | 0.97 |
|  |  | Faculty | 1-30 | 5 (3-5.7) | 3-5.8 |  |
|  | 2 | Residents | 1-20 | 10 (5-10.2) | 5-15 | 0.97 |
|  |  | Faculty | 3-30 | 7.5 (4.8-10.5) | 5-10 |  |
|  | 3 | Residents | 2-25 | 10 (5-15) | 5-15 | 0.99 |
|  |  | Faculty | 3-30 | 10 (4.9-15) | 5-15 |  |
|  | 4 | Residents | 1-50 | 10 (5-20) | 4.5-20 | 0.97 |
|  |  | Faculty | 3-50 | 12 (6.3-20) | 6.3-20 |  |

TS: teaching style, 95%-CI: 95% confidence interval, IQR interquartile range
